# Optimized Apamin-Mediated Nano-Lipidic Carrier Potentially Enhances the Cytotoxicity of Ellagic Acid against Human Breast Cancer Cells

**DOI:** 10.3390/ijms23169440

**Published:** 2022-08-21

**Authors:** Shaimaa M. Badr-Eldin, Hibah M. Aldawsari, Usama A. Fahmy, Osama A. A. Ahmed, Nabil A. Alhakamy, Omar D. Al-hejaili, Alhanoof A. Alhassan, Ghadeer A. Ammari, Shouq I. Alhazmi, Raghad M. Alawadi, Rana Bakhaidar, Abdulmohsen J. Alamoudi, Thikryat Neamatallah, Singkome Tima

**Affiliations:** 1 Department of Pharmaceutics, Faculty of Pharmacy, King Abdulaziz University, Jeddah 21589, Saudi Arabia; 2 Center of Excellence for Drug Research and Pharmaceutical Industries, King Abdulaziz University, Jeddah 21589, Saudi Arabia; 3 Mohamed Saeed Tamer Chair for Pharmaceutical Industries, King Abdulaziz University, Jeddah 21589, Saudi Arabia; 4 Department of Pharmacology and Toxicology, Faculty of Pharmacy, King Abdulaziz University, Jeddah 21589, Saudi Arabia; 5 Department of Medical Technology, Faculty of Associated Medical Sciences, Chiang Mai University, Chiang Mai 50200, Thailand

**Keywords:** nanotechnology, phospholipon, tristearin, apamin, emulsomes, ellagic acid, central composite design, cytotoxicity, breast cancer

## Abstract

Ellagic acid has recently attracted increasing attention regarding its role in the prevention and treatment of cancer. Surface functionalized nanocarriers have been recently studied for enhancing cancer cells’ penetration and achieving better tumor-targeted delivery of active ingredients. Therefore, the present work aimed at investigating the potential of APA-functionalized emulsomes (EGA-EML-APA) for enhancing cytototoxic activity of EGA against human breast cancer cells. Phospholipon^®^ 90 G: cholesterol molar ratio (PC: CH; X_1_, mole/mole), Phospholipon^®^ 90 G: Tristearin weight ratio (PC: TS; X_2_, *w*/*w*) and apamin molar concentration (APA conc.; X_3_, mM) were considered as independent variables, while vesicle size (VS, Y_1_, nm) and zeta potential (ZP, Y_2_, mV) were studied as responses. The optimized formulation with minimized vs. and maximized absolute ZP was predicted successfully utilizing a numerical technique. EGA-EML-APA exhibited a significant cytotoxic effect with an IC_50_ value of 5.472 ± 0.21 µg/mL compared to the obtained value from the free drug 9.09 ± 0.34 µg/mL. Cell cycle profile showed that the optimized formulation arrested MCF-7 cells at G2/M and S phases. In addition, it showed a significant apoptotic activity against MCF-7 cells by upregulating the expression of *p53, bax* and *casp3* and downregulating *bcl2*. Furthermore, NF-κB activity was abolished while the expression of *TNfα* was increased confirming the significant apoptotic effect of EGA-EML-APA. In conclusion, apamin-functionalized emulsomes have been successfully proposed as a potential anti-breast cancer formulation.

## 1. Introduction

Breast cancer is considered the most frequent form of malignancy in women and the leading cause of cancer mortality among women in the globe, claiming the lives of 685,000 women in 2020 [1,2]. Breast cancer was diagnosed at an estimated rate of 14 new cases per 100,000 people in women over 20 years old. These data were obtained from the National Institute of Statistics and Geography in 2020 [2]. The treatment of breast cancer is usually a multimodal approach that could possibly include surgery, radiation, chemotherapy and hormonal therapy [3]. Furthermore, the efficacy of the aforementioned therapeutic strategies is increasingly reduced; this is owed to the phenomenon of multidrug resistance (MDR) exhibited by various cancer types, and the incidence of dramatic adverse effects caused by the lack of selectivity of chemotherapeutic agents [4,5]. This situation necessitates further research into more efficient breast cancer preventive and treatment options with fewer adverse effects. 

Recently, there has been increasing evidence that substances originating from natural sources possess dual advantages of having anticancer potential with reduced side effects [6]. Many naturally originating compounds can reduce breast cancer’s aggressiveness, limit malignant cell growth and modify cancer-related pathways. Thus, several studies are currently focusing on natural and dietary substances in the hope of discovering new and more successful treatment techniques for breast cancer patients [5,7].

Ellagic acid (EGA), a member of the ellagitannin family, was discovered in various berries, including strawberries, cranberries, blackberries, raspberries and goji berries, as well as in grapes, pomegranates, nuts, green tea, and Eucalyptus globulus’s stem and bark [8,9]. It is a natural phenolic component present in free and glycosylated forms, or as complex polymers esterified with a sugar known as ellagitannins [10]. Owing to its antioxidant and antiproliferative characteristics, EGA has attracted increasing attention in the medical field with versatile therapeutic applications [11]. It has been successfully applied for alleviating inflammation, treatment and protection against cardiovascular diseases and neurodegenerative disorders [12,13]. Moreover, it has demonstrated anti-aging, antiviral and antimicrobial activity [14,15]. Recently, EGA has drawn an immense attention for the prevention and management of malignancy [16]. Despite these merits, EGA suffers from drawbacks, including poor aqueous solubility (9.7 µg/mL), that could result in poor biological performance and limited clinical use [17,18]. Implementing nanotechnology for the development of EGA formulation could offer a viable means to surpass the previously mentioned hurdles.

Various nanocarriers, including lipid-based, polymer-based and inorganic-based ones, are nowadays utilized in cancer therapy for achieving enhanced solubility, anticancer activity and targeted delivery [19,20]. Because of their lipophilicity, lipid-based nanocarriers have a higher capacity to overcome biological barriers than other nanocarriers [21]. Furthermore, they are appealing due to their well-established biodegradability, biocompatibility and their capability to encapsulate both hydrophilic and hydrophobic molecules [22]. 

Emulsomes (EML) are novel lipid-based nanocarriers made up of solid lipid core surrounded by a phospholipid bilayer [23]. They combine the benefits of nanoemulsion formulations as well as liposomes [24]. A key feature of EML is the existence of the lipid core as a solid or liquid crystalline state instead of oil in a liquid state. Interestingly, this allows EML to entrap larger amounts of lipid-soluble medicines with a more sustained release profile [25]. The outermost structure of EML, the phospholipid bilayer, eliminates the need for stabilization by surfactant; this feature offers a high level of stability and biocompatibility with minimized toxicity. The phospholipid sheath also imparts the ability to encapsulate the hydrophilic molecules in the aqueous parts of the surrounding phospholipid layers and/or load the lipophilic molecules into their inner core [24,26]. Furthermore, the nanosize of the formulation might substantially increase the emulsomal dispersions’ drug targeting impact [27]. Because of these features, EML might be regarded as promising stable surfactant-free alternatives to the first generation lipid-based nanocarriers that could safely and efficiently deliver various drugs [28].

Various approaches have emerged for surface modification of nanocarriers used in cancer therapy with the aim of prolonging circulation time, enhancing cancer cells penetration and achieving better tumor-targeted delivery [29]. Amongst these approaches, conjugation with peptides has recently attracted great attention. Apamin (APA), a natural toxin, accounting for approximately 2–3% of the bee venom (BV) dry weight, is a small peptide which is structurally made of 18 amino acids [30]. It has many pharmacological effects which could be tailored for many key therapeutic, including anti-inflammatory, anti-fibrotic and anti-atherosclerotic actions [31,32]. In addition, it has been successfully applied in targeted drug delivery [33]. It is worth noting that cytotoxic activity has been reported for bee venom components [31]. 

Therefore, the present work aimed at exploring the potential of APA-functionalized emulsomes (EGA-EML-APA) as a peptide-mediated vesicular delivery approach for enhancing cytotoxic activity of EGA against human breast cancer cells. Central composite response surface design was implemented for optimization of the proposed emulsomal formulation with minimized vesicle size (VS) and maximized absolute zeta potential (ZP). The optimized formulation was tested for cytotoxic and proapoptotic activity against human breast cancer cells.

## 2. Results and Discussion

Several nano-sized formulations such as liposomes, nanoemulsions, niosomes, proniosomes, nanoparticles and ethosomes have gained a lot of attention in the field of drug delivery. Nevertheless, emulsomes possess unique characteristics that impart potential advantages to this system compared to others. The most unique characteristic of emulsomes, compared to other vesicular systems including liposomes and niosomes, is the solid fat core enclosed by phospholipid. This allows for enhancing the solubility of poorly soluble drugs and entrapping high amounts of such drugs within the core. The enclosed drug exhibits prolonged drug release and consequently extended efficacy. Second, an emulsomes-based system showed adequate potential for targeting by virtue of their nano-size. Another reported advantage is surpassing the development of multi drug resistance, often associated with over expression of a cell membrane glycoprotein, which causes efflux of the drug from the cytoplasm and results in an ineffective drug concentration inside the cellular compartment [34]. Accordingly, emulsomes were chosen for investigation in this work. In addition, apamin was used for surface-functionalization of the optimized mulsomal formulation to provide additional advantage of enhancing uptake of EGA by cancerous cells [35]. It is worth noting that the reported cytotoxic activity of bee venom components including apamin could also augment the cytotoxicity of EGA [31]. 

### 2.1. Face-Centred Central Composite Design Analysis

#### 2.1.1. Model Fit Statistics

Fit statistical analysis results for the responses, namely, vs. and ZP are presented in Table 1. Based on the highest R^2^ and the least PRESS, the vs. data fitted the quadratic model, while the ZP fitted the two-factor interaction (2FI) model. The adjusted R^2^ and the predicted R^2^ for each response exhibited appropriate coincidence with a difference of less than the permissible limit of 0.2 verifying the model validity. Moreover, the selected model for each response exhibited adequate precision value higher than the desirable value of 4 indicating appropriate signal-to-noise ratio. According to the previously computed parameters, the selected models could be adequately utilized for the exploration of the experimental design space.

**Figure 1:**
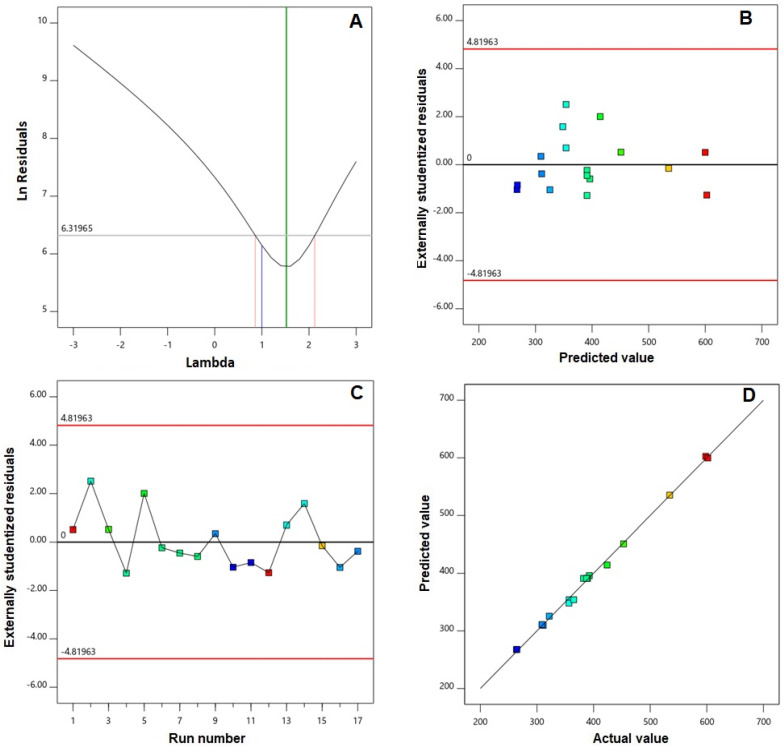
Diagnostic plots for vesicle size of EGA-EML-APA. (**A**) Box–Cox plot for power transforms (**B**) externally studentized residuals vs. predicted values plot (**C**) externally studentized residuals vs. run number plot and (**D**) predicted vs. actual values plot. Abbreviations: EGA, ellagic acid; EML, emulsomes; APA, apamin.

**Figure 2:**
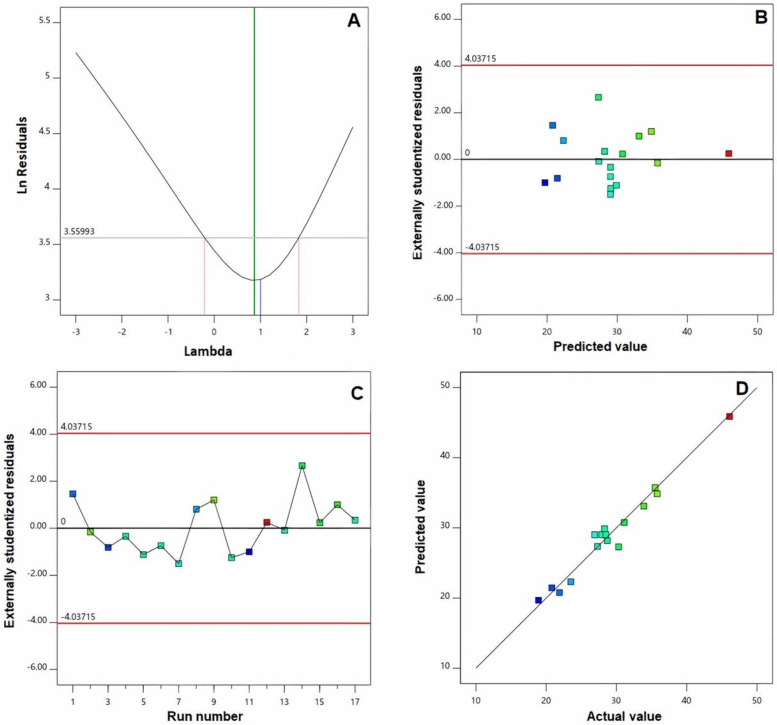
Diagnostic plots for zeta potential of EGA-EML-APA. (**A**) Box–Cox plot for power transforms (**B**) externally studentized residuals vs. predicted values plot (**C**) externally studentized residuals vs. run number plot and (**D**) predicted vs. actual values plot. Abbreviations: EGA, ellagic acid; EML, emulsomes; APA, apamin.

Furthermore, verification of the goodness of fit of selected models was carried out via developing diagnostic plots illustrated in Figure 1 and Figure 2. Box–Cox plot for power transforms, seen in Figure 1A and Figure 2A, show the best lambda (λ) value of 1.52 and 0.87 (represented by the green line) for vs. and absolute ZP, respectively. The computed confidence intervals (represented by the red lines) around these lambdas comprise the current λ value of 1 (represented by the blue line); therein, no specific transformation for observed vs. is suggested [28]. The computed maximum to minimum measured vs. and absolute ZP ratio of 2.28 and 2.44, respectively, supports the absence for necessity of transformation, where a ratio exceeds 10 calls for a transformation requirement. Studentized residual is a good criterion for identifying potential outliers that could influence the regression model. In our study, the colored dots that symbolize the measured vs. and absolute ZP in the externally studentized residuals vs. predicted response plot, Figure 1B and Figure 2B, shows randomly scattered points within the boundaries implying the absence of constant error. In addition, the colored points in the externally studentized residuals vs. run plots, and Figure 1C and Figure 2C show that no lurking variable could influence the determined responses as evidenced by random scatter of points and absence of trends that could possibly indicate a time-related variable lurking in the background. Additionally, the predicted versus actual plots, illustrated in Figure 1D and Figure 2D showed a highly linear pattern, indicating that the observed responses are analogous to the corresponding predicted values [36,37].

#### 2.1.2. Influence on vs. (Y_1_)

Invading cancerous tissues is considered a significant problem in the development of formulations for moieties with anticancer activity. This needs ongoing research to alter the features of drug delivery systems in order to improve tumor accessibility [38]. Because of their preferential distribution within solid malignant masses, nanoparticulate formulations of average sizes being less than 400 nm have lately gained an enormous amount of attention in the arena of malignant tumors therapy [39,40]. Despite this discovery, it has been reported that ineffective tumor tissue penetration, resulting from the pathological situation generated by malignancy progression, could overcome the preferential accumulation of nano-sized delivery systems within the malignant tissues and their related therapeutic efficacy [41]. Accordingly, it is possible to verify tumor penetration enhancement by lowering the size to the smallest achievable value, which in turn leads to a higher surface area available for penetrating the tissues [42]. Accordingly, minimized size was set as a goal to increase surface area available for permeation, and thus to ensure effective tumor penetration. 

In our study, the VS of the prepared EML ranged between 263.7 ± 7.9 and 601.8 ± 18.9 nm (Table 2). On the basis of analysis of variance (ANOVA) provision for VS, the significance of the quadratic model was confirmed as evidenced by the F-value of 265.35 (*p* = 0.0001). The lack of fit F-value of 6.32 (*p* = 0.1448) shows a non-significant lack of fit; thus, fitting of the observed vs. to the recommended model is ensured. The equation demonstrating the quadratic model in terms of coded factor was generated by the software is given as:

Y_1_ (vesicle size) = 391.15 + 93.50 X_1_ + 51.69 X_2_ + 20.95 X_3_ + 58.71 X_1_X_2_ + 13.69 X_1_X_3_ − 36.04 X_2_X_3_ + 50.68 X_1_^2^ − 28.47 X_2_^2^ − 16.07 X_3_^2^

**Figure 3:**
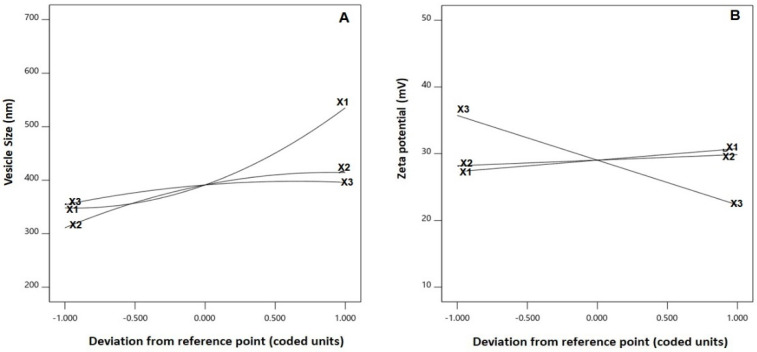
Perturbation graph for the main effects of critical attributes; PC: CH molar ratio (X_1_), PC: TS weight ratio (X_2_) and APA molar concentration (X_3_) on (**A**) vesicle size and (**B**) zeta potential of EGA-EML-APA. Abbreviations: EGA, ellagic acid; EML, emulsomes; APA, apamin; PC: Phospholipon 90 G; Cholesterol: CH; TS; Tristearin.

**Figure 4:**
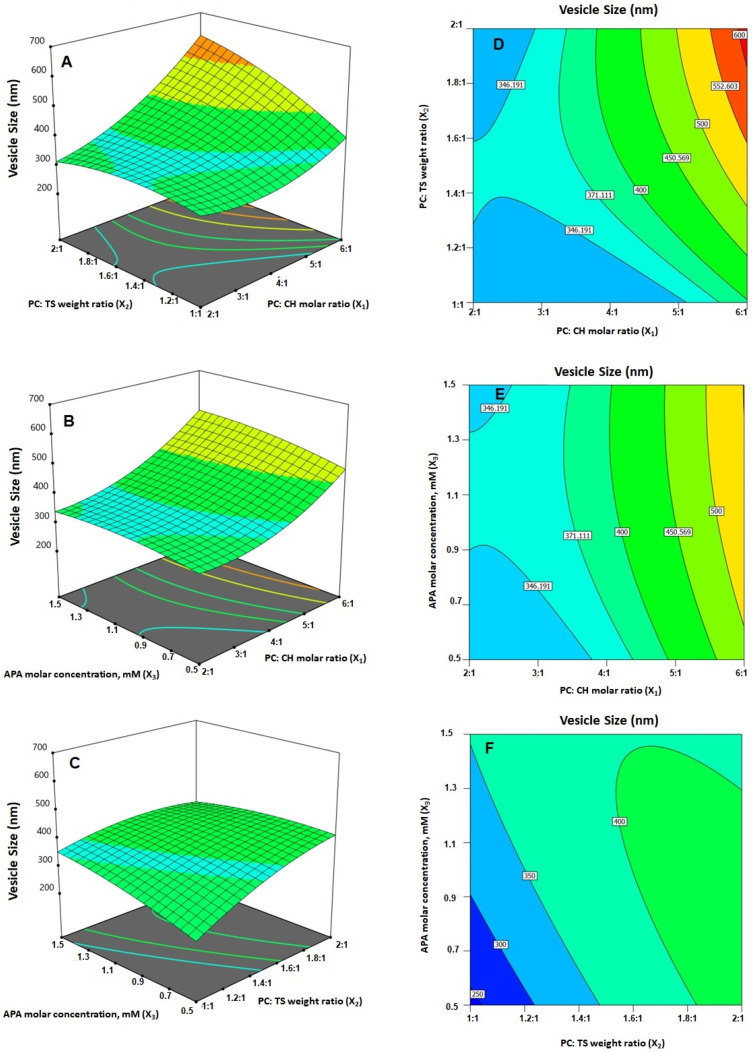
(**A**–**C**) Response surface 3D- and (**D**–**F**) contour 2D-plots showing the interaction between the investigated variables on the vesicle size of EGA-EML-APA. Abbreviations: EGA, ellagic acid; EML, emulsomes; APA, apamin; PC: Phospholipon 90 G; Cholesterol: CH; TS; Tristearin.

As per ANOVA results, all the linear terms belonging to the analyzed factors had a significant effect on the emulsomal vs. (*p* < 0.0001 for all terms). Furthermore, the interaction terms as well as the quadratic terms were also significant at the 95% level of significance. The perturbation graph, displayed in Figure 3A, demonstrates the influence of the investigated factors on the VS, while the 3D-response and the 2D-contour plots, displayed in Figure 4, demonstrate the interaction between them. The illustrations show that the EML vs. significantly increases at higher PC: CH molar concentration, PC: TS weight ratio and APA molar concentration. Clearly, the positive sign associated with the linear term coefficients; X_1_, X_2_ and X_3_ supports this finding. The most influential factor was PC: CH molar ratio as indicated by the highest coefficient of its linear term X_1_ in the developed equation, followed by PC: CH molar ratio, then the APA molar concentration. 

The effect of PC: CH ratio could be attributed to the possible creation of numerous bilayers, which results in greater vs. [25]. Nonetheless, a comparable increase in vs. of zidovudine EML was previously reported upon increasing the phospholipid relative to the solid lipid and cholesterol. On the other hand, the VS was inversely proportional to the level of cholesterol. In fact, given that, at increased cholesterol levels, the EML acquires a stronger lipophilic property, which encourages inhibition of water uptake across the lipid bilayer; this explains the observed reduction in VS. This observation is consistent with Sudhakar and Chaitanya’s [43] work, who found an inverse association between vs. and cholesterol level of ritonavir liposomes.

#### 2.1.3. Influence on Zeta Potential (Y_2_)

The surface charge of the vesicles is described by ZP, which reflects their physical stability, where higher surface charge results in electrostatic repulsive forces that prevent particles coalescence and aggregation. Generally, nano-formulations with ZP values of about or greater than ±30 mV are considered stable [23]. The prepared EGA-EML-APA exhibited negative ZP values ranging from −18.9 ± 0.6 to −46.1 ± 1.9 mV, Table 2. The negative charge could be credited to the negatively charged phospholipid that forms the lipid bilayer surrounding emulsomal core. It is worth noting that, although a negatively charged surface might slightly affect the cellular internalization, the stability of nano-systems against aggregation is crucial for benefiting from the nano-size. In addition, many studies have reported enhanced anticancer activity for negatively charged nanoformulation [44,45,46,47]. Accordingly, the study aimed at maximizing the absolute ZP value.

Based on Analysis of variance (ANOVA) for absolute ZP, the significance of the two-factor interaction model was confirmed, as depicted by the corresponding F-value of 44.69 (*p* < 0.0001). The lack of fit F-value of 4.37 (*p* = 0.1997) reflects non-significant lack of fit; thus, the fitting of absolute ZP values to the proposed model is confirmed. The equation generated by the software demonstrates the suggested model for the ZP in terms of coded factor as given below:

Y_2_ (zeta potential) = 29.03 + 1.72 X_1_ + 0.84 X_2_ − 6.70 X_3_ + 1.75 X_1_X_2_ − 2.92 X_1_X_3_ − 2.92 X_2_X_3_

**Figure 5:**
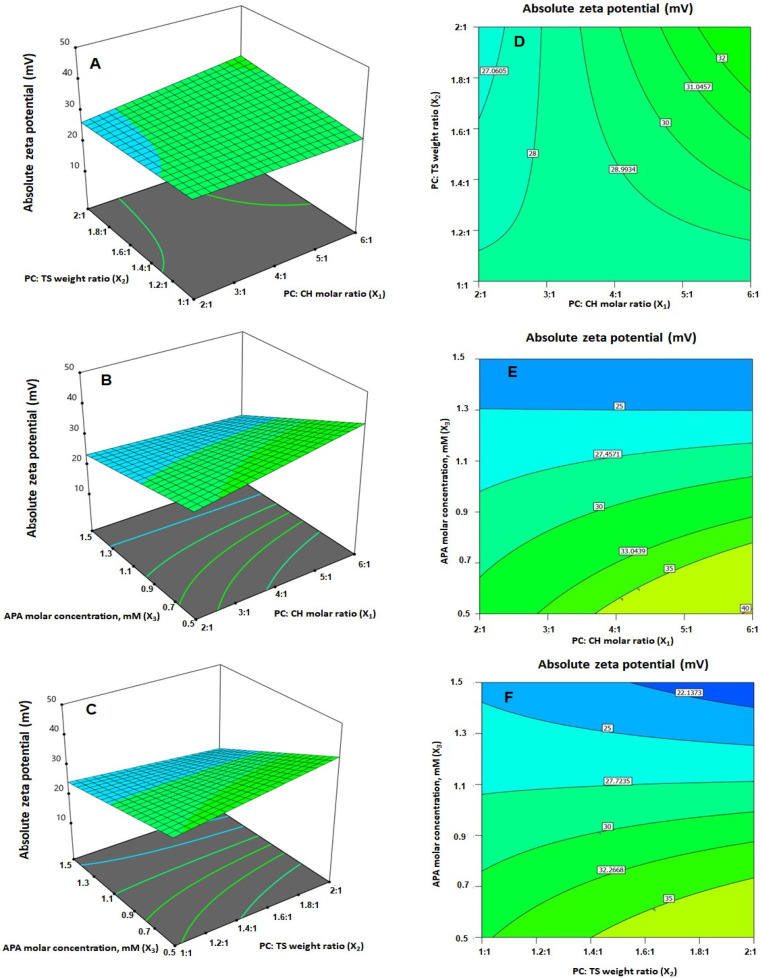
(**A**–**C**) Response surface 3D- and (**D**–**F**) contour 2D-plots showing the interaction between the investigated variables on the absolute zeta potential value of EGA-EML-APA. Abbreviations: EGA, ellagic acid; EML, emulsomes; APA, apamin; PC: Phospholipon 90 G; Cholesterol: CH; TS; Tristearin.

The statistical analysis demonstrated that the linear terms X_1_ and X_3_ corresponding to PC: CH molar ratio and APA molar concentration exhibited a significant effect on absolute ZP (*p **=*** 0.0057 for X_1_ and *p* < 0.0001 for X_3_). Additionally, all the interaction terms were significant at *p* < 0.05. Figure 3B depicts the perturbation graph that demonstrates the main effects of the investigated variables on the absolute ZP, while Figure 5 illustrates the 3D-surface plots and the 2D contour plots that show the interaction between the variables. The illustrations reveal higher ZP absolute values, with considerable increase in the negativity of the emulsomal surface, which were observed when the PC: CH molar ratio was increased. On the other hand, the absolute ZP decreases at higher APA concentrations. The positive sign of X_1_ and the negative sign of X_3_ coefficients support this observation. The observed interlink between increased negativity with the concomitant increase in PC: CH molar ratio could probably be a result of an increase in the proportion of a negatively charged phospholipid in the outer layers [3,21]. On the contrary, the observed reduced negativity at higher APA molar concentration could be credited to the possible interaction between the positively charged lysine moieties present in APA structure with the negatively charged phospholipid of the emulsomal vesicles.

### 2.2. Optimization of EGA-EML-APA

**Figure 6:**
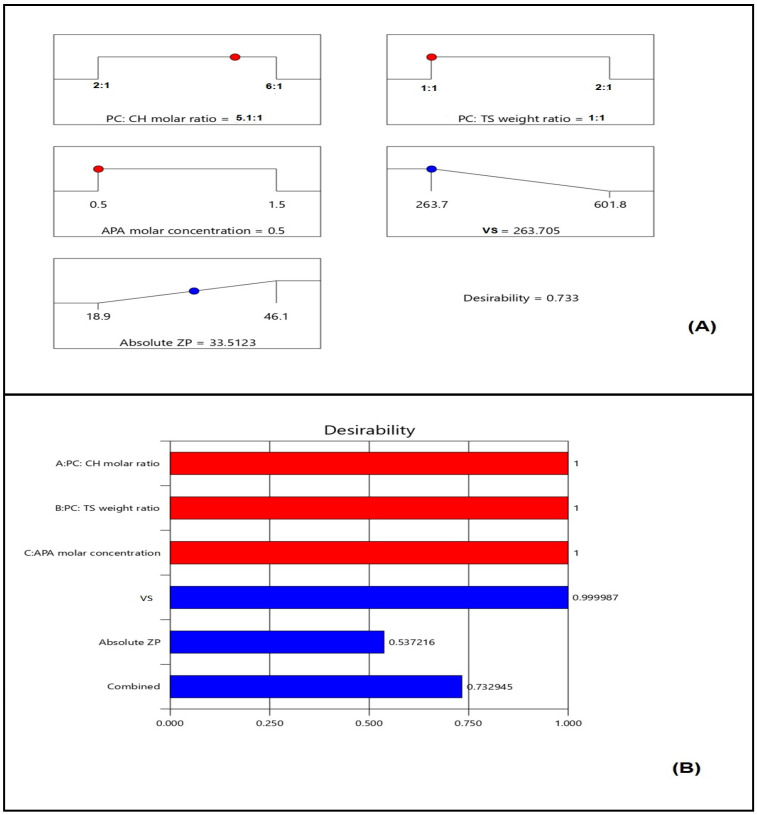
(**A**) Ramp graphs representing the optimized levels of the independent variables and the predicted responses for the optimized EGA-EML-APA (Red points represent the optimized levels of variables and blue points represent predicted response); (**B**) desirability values for the predicted responses and overall desirability of the optimized ICA tochozeinolate nanospheres. Abbreviations: EGA, ellagic acid; EML, emulsomes; APA, apamin; PC: Phospholipon 90 G; Cholesterol: CH; TS; Tristearin. Red points represent the optimized levels of variables and blue points represent predicted response.

Numerical optimization and the desirability techniques were implemented for predicting the optimized variables’ levels that, upon combination, could result in minimized vs. and maximized absolute ZP. The ramp graphs presented in Figure 6A depict the optimized levels and the predicted responses, while the desirability for each response and the overall desirability are graphically illustrated in Figure 6B. The measured vs. of 267.6 nm and absolute ZP of 32.1 mV coincide well with the predicted ones showing relative percentage error of 1.47% and 4.17%, respectively. Furthermore, the measured vs. is less than 400 nm indicating adequate preferential distribution within solid cancerous tissues [39,40], and the absolute ZP is greater than 30 mV, indicating adequate repulsive forces that guard against aggregation [23]. The relatively small computed percentage errors prove the reliability of the optimization process. It is worth noting that the PS and ZP values of the optimized formulation are in good agreement with those reported previously for other nanocarriers with enhanced cytotoxicity against various cancer cells. Barani et al. reported enhanced anticancer activity for paclitaxel loaded niosomes with an average size of 240 nm [48]. The reported size was sufficient for tumor-specific accumulation. Abbas et al. reported an enhanced cytotoxic effect for optimized curcumin bilosomes with maximized negative absolute zeta potential of −27.05 mV [46]. On the other hand, previous studies that involve vesicles with higher size in the micro range exhibited low cytotoxicity profiles on different cell lines. Lai et al. developed oleic acid containing vesicles with an average size of 2.35 ± 0.9 μm for transdermal delivery [49]. Cytotoxicity studies of such vesicles in various skin cell lines demonstrated no loss of cell viability in all concentrations indicating high safety profile and appropriateness for transdermal use with no side effects on the skin. However, the diminished cytotoxicity of the micro-sized vesicles could indicate poor ability to accumulate within the cells, thus highlighting the benefit of the nano-size in our study. This indicates the importance of developing a carrier to improve delivery of loaded active ingredients [50].

### 2.3. Characterization of Optimized EGA-EML-APA

**Figure 7:**
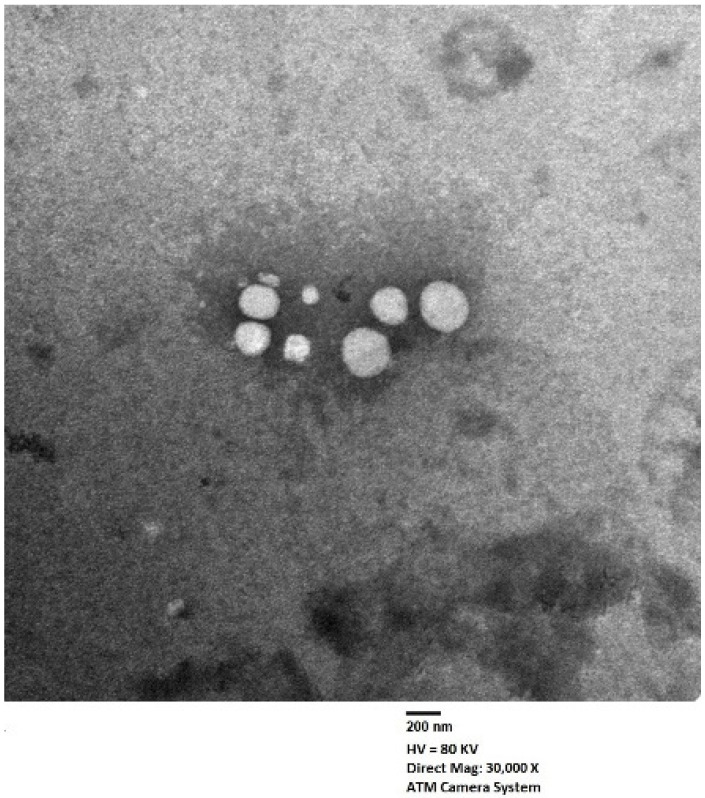
Transmission electron micrograph of optimized EGA-EML-APA. Abbreviations: EGA, ellagic acid; EML, emulsomes; APA, apamin.

The shape of the optimized EGA-EML-APA was visualized using TEM as illustrated in Figure 7. The TEM micrograph reveals spherical vesicles that possess even rounded contours and adequate uniform distribution. The average size of the vesicles is in good agreement with that measured by the dynamic light scattering technique. Zhou and Chen [23] reported a similar spherical shape for silybin nanoemulsomes.

The optimized formulation showed high entrapment efficiency of 93.7 ± 4.1%. The high entrapment ability of the proposed formulation could be due to the solid lipid core enclosed by the lipid bilayer which aids in increasing the entrapping of poorly soluble active ingredients [34]. It is worth noting that this feature contributes to the superiority of emulsomes over other vesicular carriers including niosomes; for example, pH-responsive niosomes showed entrapment of less than 80% for paclitaxel in a previous study [48]. 

### 2.4. EGA-EML-APA Inhibited the Viability of MCF-7 Cells

**Figure 8:**
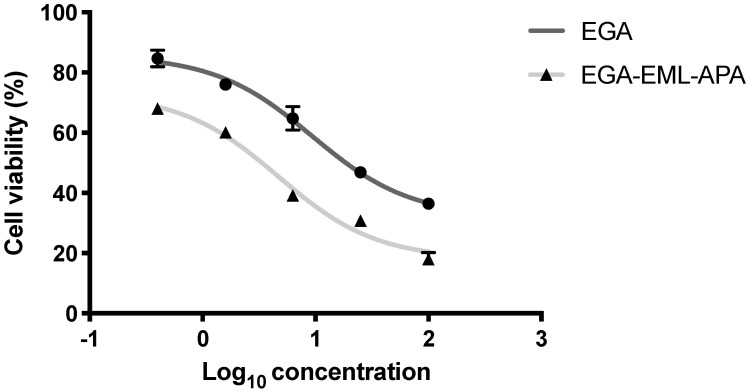
Cell viability evaluation using the MTT assay after 24 h of treatment with EGA and EGA-EML-APA. Data are expressed as mean ± SD. Abbreviations: EGA, ellagic acid; EML, emulsomes; APA, apamin.

Breast cancer is the most commonly occurring cancer in women and the second most common cancer overall, specifically estrogen receptor positive (ER) breast cancer. Ellagic acid has been demonstrated to have antitumor and anti-apoptotic effects in several cancer cells [8]. However, in MCF-7, the anti-proliferative effect of ellagic acid was observed in only high concentrations [6,51,52]. Therefore, MCF-7 cells were selected to examine the anti-proliferative effect of the optimized EGA-EML-APA formula. EGA-EML-APA was found to significantly inhibit MCF-7 cell viability, and in a manner that seems to be dose-dependent, more than EGA. As shown in Figure 8, the IC_50_ value for EGA-EML-APA in MCF-7 was 5.472 ± 0.21 µg/mL compared to 9.09 ± 0.34 µg/mL for EGA. Furthermore, there was no effective cytotoxic effect of blank EML-APA when compared with the EGA and EGA-EML-APA in MCF-7 cells. The observed cytotoxic activity of the EGA-EML-APA is in line with the previously reported cytotoxicity of EGA against breast cancer cells including MCF-7 cells [6,53]. However, EGA-EML-APA showed significantly improved cytotoxic activity against MCF-7 cells compared to EGA. This promising effect may be to the result of the enhanced cellular penetration and accumulation of EGA in MCF-7 cells by virtue of the optimized formulation. The ability of EML to enhance cellular uptake of actives has been previously reported [54,55,56].

### 2.5. EGA-EML-APA Modulated MCF-7 Cell Cycle 

**Figure 9:**
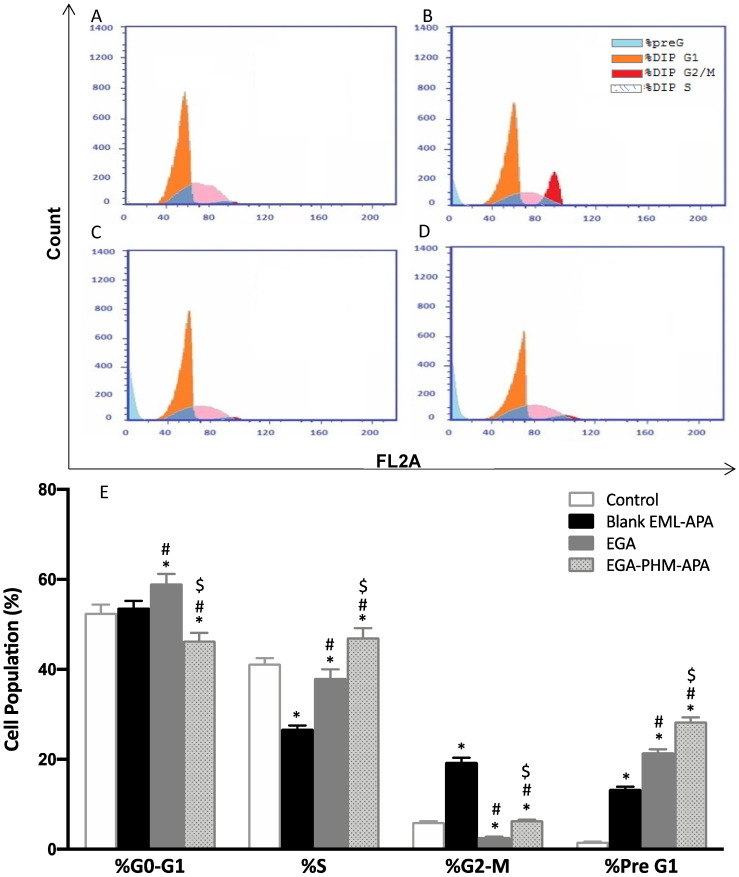
Flow cytometric analysis of MCF-7 control cells (**A**), or treated with blank EML-APA (**B**), EGA (**C**) and EGA-EML-APA (**D**) and the cellular fractions in the phases of the cell cycle (**E**). Data are expressed as mean ± SD. * Significantly different from the control at *p* < 0.05, # significantly different from blank EML-APA at *p* < 0.05. $ Significantly different from EGA at *p* < 0.05. Abbreviations: EGA, ellagic acid; EML, emulsomes; APA, apamin.

To determine the mechanism by which EGA-EML-APA exerts its cytotoxic influence against MCF-7 cells, the effect on cell cycle phases was investigated. While untreated MCF-7 cells exhibited quick growth properties, treatment of the cells with the EGA, and EGA-EML-APA resulted in altered cell cycle progression and increased cell fraction in the pre-G1 phase (Figure 9C–E). The percentages of cells accumulated in the pre-G1 phase were 21.57 ± 0.69% and 28.42 ± 0.94% when cells were treated with EGA and EGA-EML-APA, respectively. As can be seen from Figure 9E, the most significant increase in cell fraction of the pre-G1 phase was associated with EGA-PHM-APA. Additionally, EGA-EML-APA caused a marked reduction in cell population in G0/G1 phase in comparison with other groups (*p* < 0.05) (Figure 9C,E). Furthermore, the fractions of cell in G2/M and S phases significantly increased when compared to the control and EGA (*p* < 0.05). These data suggest that EGA-EML-APA caused sequential cell cycle arrest at G2/M and S phases, followed by apoptosis, which is represented by the increase in apoptotic pre-G1 population. A previous report demonstrated a comparable ability of EGA to interfere with the cell cycle especially by increasing the population of cells at the pre-G1 phase [57]. However, treatment with EGA-EML-APA resulted in a significant increase in the S, G2-M and pre-G1 phases compared to EGA. Hence, the abovementioned results highlight the improved cytotoxic activity of the optimized formulation. 

### 2.6. EGA-EML-APA Induced Apoptosis in MCF-7 Cells

**Figure 10:**
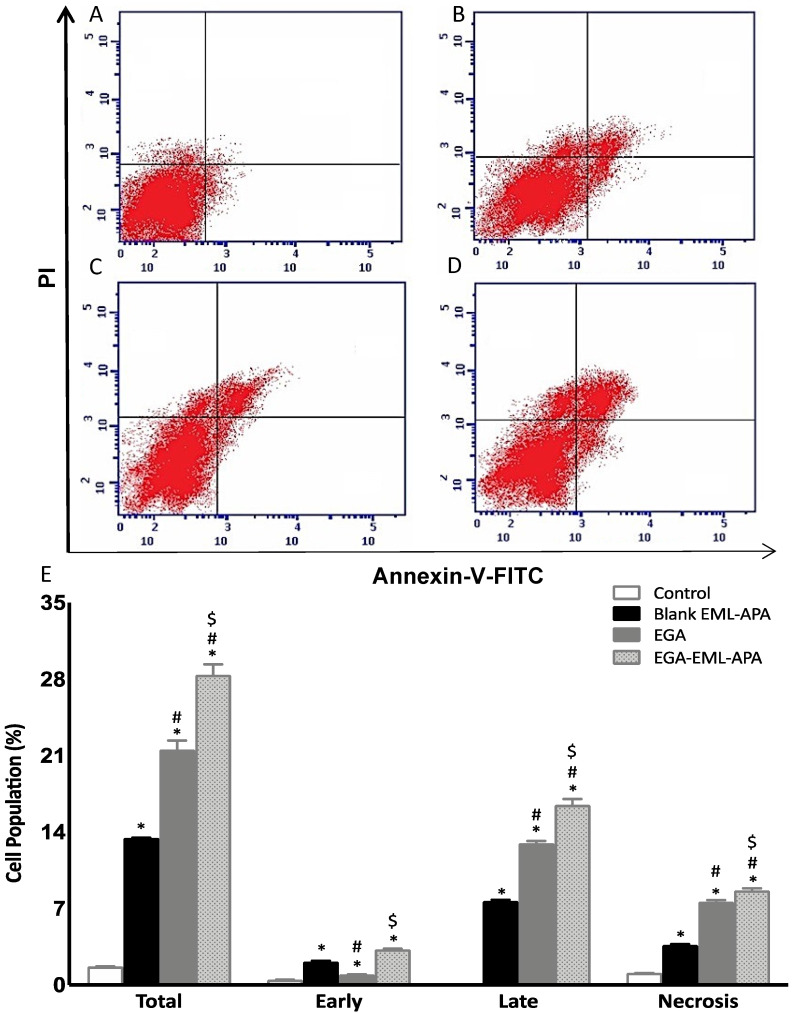
Assessment of apoptosis in MCF-7 control cells (**A**), or treated with blank EML-APA (**B**), EGA (**C**) and EGA-EML-APA (**D**) and the percentages of early and late apoptotic cells (**E**) following annexin V staining. Data are expressed as mean ± SD. * Significantly different from control at *p* < 0.05, # significantly different from blank EML-APA at *p* < 0.05, $ significantly different from EGA at *p* < 0.05. Abbreviations: EGA, ellagic acid; EML, emulsomes; APA, apamin.

The increased cellular fraction in the pre-G1 phase strongly indicates that the optimized EGA-EML-APA exhibited a significant apoptotic activity. Thus, annexin-V staining was utilized to determine the percentages of apoptotic cells associated with blank EML-APA, EGA and EGA-EML-APA. As can be seen from Figure 10B,C,E, treating MCF-7 cells with EGA-EML-APA formula resulted in the most significant increase in early, late and total apoptotic cell death when compared to EGA (*p* < 0.05). Necrotic cell death showed a similar pattern in which the most significant increase was associated with EGA-EML-APA (Figure 10C,E). This apoptotic effect produced by EGA-EML-APA could be due to the ability of EGA to alter the expression of cellular components involved in apoptosis and cell-cycle regulation. Therefore, the expression of apoptosis markers involved in the intrinsic mitochondrial mediated apoptosis pathway such as *bax*, *bcl-2*, *p53* and *casp-3* is investigated. 

### 2.7. EGA-EML-APA Apoptotic Effect Evidenced by MMP Loss and Apoptotic Markers

**Figure 11:**
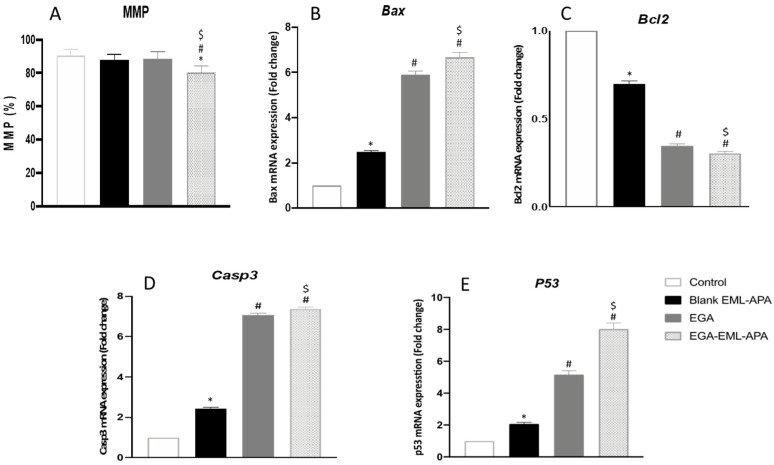
Effect of the EGA-EML-APA on MMP in MCF-7 cells. (**A**), the mRNA expression on *Bax* (**B**), *Bcl2* (**C**), *Casp3* (**D**), *P53* (**E**). Data are presented as Mean ± SD (n = 3). *, # or $: Statistically different from control, blank EML-APA or EGA, respectively at *p* < 0.05. Abbreviations: EGA, ellagic acid; EML, emulsomes; APA, apamin.

Upon exposure to apoptotic stimuli, damage to the mitochondrial membrane might occur, resulting in the loss of MMP [58]. Hence, the change in MMP was measured in MCF-7 control cells, and after adding blank EML-APA, EGA or EGA-EML-APA. As shown in Figure 11A, MMP was significantly compromised only when the cells were treated with the optimized EGA-EML-APA (*p* < 0.05), leading to a significant loss of MMP in comparison to raw EGA. Thus, an enhanced efficacy with EGA-EML-APA could be obviously shown and can be attributed to the lipophilic nature of the delivery system, which plays a major role in enhancing the delivery of EGA incorporated into this novel nanocarrier platform [59].

The effect of ellagic acid through the intrinsic apoptotic pathway in MCF-7 cells was successfully reported in several works [51,52,60]. The expression of multiple markers of this pathway including *Bax*, *Bcl2* and *casp3* was also examined to confirm the apoptotic activity of EGA-EML-APA. As depicted in Figure 11B, Bax mRNA was significantly higher in EGA-EML-APA-treated cells relative to EGA. In contrast, EGA-EML-APA was associated with the lowest expression level of *Bcl2*, Figure 11C. Additionally, MCF-7 cells treated with EGA-EML-APA exhibited the most significant increase in *casp3* expression compared to EGA treatment (*p* < 0.05), Figure 11D. These findings highlight the superior activity of the EGA-EML-APA in apoptosis induction relative to EGA, considering that increased *Bax* levels and decreased Bcl-2 levels favor apoptosis [61]. It was reported that EGA exhibits potent apoptotic activities by enhancing the expression of *casp3*, which is an apoptotic transcription factor [62]. In this regard, EGA-EML-APA treatment increased the expression of *p53* by 8-fold compared to 5-fold increases associated with EGA (Figure 11E). This further confirms that EGA-EML-APA significantly improved the cytotoxicity and apoptotic activity EGA.

### 2.8. EGA-PHM-APA Induced Changes in the Expression of tnf-α and Nf-κb 

**Figure 12:**
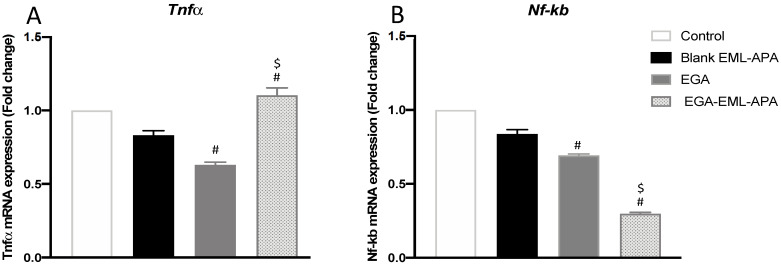
Effect of the optimized EGA-EML-APA formula on the expression of *tnf-α* (**A**) and *nf-κb* (**B**) in MCF-7 cells. Data are presented as Mean ± SD (n = 3). # or $: Statistically different from blank EML-APA or EGA, respectively, at *p* < 0.05. Abbreviations: EGA, ellagic acid; EML, emulsomes; APA, apamin.

TNF-α has been utilized as an inflammatory marker in wound healing studies [63]. It has also been explored as an apoptotic marker; it is reported that TNF-α can induce apoptosis in breast cancer cells, and this process can be inhibited upon NF-κB activation [64,65]. As shown in Figure 12A, greater levels of TNF-α were detected with EGA-EML-APA treatment. In addition, EGA-EML-APA produced the most significant decrease in NF-κB activation (*p* < 0.05) (Figure 12B). Therefore, these findings further substantiate the superior cytotoxic activity of EGA-EML-APA compared to the blank EML and EGA.

## 3. Materials and Methods

### 3.1. Materials

Ellagic acid (EGA), Cholesterol (CH), Tristearin (TS) and Apamin (APA) were purchased from Sigma-Aldrich Inc. (St. Louis, MO, USA). Phospholipon^®^ 90 G (Purified Phosphatidylcholine from Soybean Lecithin, content 90%, PC) was obtained as a gift sample from Lipoid GmbH (Ludwigshafen, Germany).

### 3.2. Response Surface Methodology for Formulation of EGA-EML-APA

Response surface methodology was implemented for the formulation and optimization of EGA-EML-APA. Specifically, three-level, three-factor, face-centred central composite experimental design (α = 1) was utilized in this study. Phospholipon^®^ 90 G: cholesterol molar ratio (PC: CH; X_1_, mole/mole), Phospholipon^®^ 90 G: tristearin weight ratio (PC: TS; X_2_, *w*/*w*) and APA molar concentration (APA conc.; X_3_, mM) were considered as numerical independent variables. Vesicle size (VS, Y_1_, nm) and zeta potential (ZP, Y_2_, mV) were investigated as responses. The coded levels of the independent variables, represented as –1, 0, +1 analogous to lower, middle and upper values respectively, along with the corresponding actual values for each variable are listed in Table 3. Design-Expert software (Version 12; Stat-Ease Inc., Minneapolis, MN, USA) was used to generate the experimental runs. As per the selected design, 17 runs were generated including factorial points, axial points and three replicated centre points to give appropriate prediction ability close to the centre of the variable space; the variables’ levels represented in each run are compiled in Table 2. The sequential model that best fits the data of each response was determined according to the predicted and adjusted determination coefficients (R^2^), as well as the predicted residual error sum of squares (PRESS) statistics. The equations expressing the optimal model for the responses were then developed by the software. The significance of the variables on the measured responses was analyzed using analysis of variance (ANOVA) at *p* < 0.05. Perturbation and response surface plots were developed to display the influence of the explored factors as well as their interactions.

### 3.3. Optimization of EGA-EML-APA

A numerical optimization technique following a desirability approach was used for prediction of the optimized EGA-EML-APA formulation; the desirability function amalgamates both responses aiming at anticipating the optimized levels of investigated factors [66,67]. The objective of the study was achieving minimized vs. and maximized ZP absolute value as shown in Table 1.

### 3.4. Preparation of EGA-EML-APA

A previously reported method was utilized for the preparation of EGA-EML-APA [26]. Accurately weighed quantities of EGA (50 mg), Phospholipon^®^ 90 G, cholesterol and tristearin were solubilized in 15 mL of chloroform/methanol blend (2:1, *v*/*v*). Evaporation of the solvent blend was done under reduced pressure using Rotavapor (BÜCHI Labortechnik AG, Flawil, Switzerland) at 40 °C. By placing the formed film in a vacuum oven for 24 h, any organic solvent residual was eliminated. Hydration of the films was then achieved by mild agitation with 10 mL distilled water containing APA for 3 h at room temperature. The resultant dispersion was ultra-sonicated for two cycles, each of 45 s with a time gap of two minutes for vs. reduction [25,68]. 

### 3.5. Vesicle Size and Zeta Potential Determination

Mean vs. (z-average) as well as ZP of the proposed EGA-EML-APA were determined by light scattering and electrophoretic techniques, respectively, using Nano ZSP (Malvern Panalytical, UK) at 25 ± 1 °C. Adequate dilution with distilled water was done prior to measurement. Data were expressed as the mean of five determinations. 

### 3.6. Characterization of Optimized EGA-EML-APA

The optimized EGA-EML-APA were visualized using a JEOL GEM-1010 (JEOL Ltd., Akishima, Tokyo, Japan) transmission electron microscope (TEM) at 80 kV. One drop of diluted formulation was placed on a carbon-coated grid, which was then left to dry at temperature of 25 ± 0.5 °C. Furthermore, the sample was negatively stained with 1% phosphotungstic acid and then dried for 20 min at room temperature before being visualized. 

Entrapment efficiency of the optimized formulation was performed by an indirect method. In addition, 1 mL of emulsomes dispersion was ultracentrifuged at 100,000 rpm for 1 h at 4 °C. The residue was washed twice with phosphate buffer (pH 6.8) and subjected to re-centrifugation for 1 h. The combined supernatant was diluted with phosphate buffer (pH 6.8), and analyzed using the reported HPLC method of assay with UV detection at 254 nm. The procedure was repeated thrice [15]. 

The entrapment efficiency was calculated using the following equation:

EE% = EGA_t_ − EGA_u_/EGA_t_ × 100

where EGA_t_ represents the amount of total ellagic acid; and EGA_u_ represents the amount of unentrapped ellagic acid. 

### 3.7. In Vitro Antitumor Activity of Optimized EGA-EML-APA on MCF-7 Cells

#### 3.7.1. Cell Culture

Human breast cancer cell line (MCF-7) was obtained from the American Type Culture Collection (Manassas, VA, USA). The cells were cultured in Dulbecco’s Modified Eagle Medium (DMEM) containing 10% FBS, penicillin and streptomycin at a concentration of 1% *v*/*v* in a humidified atmosphere with 5% CO_2_ incubator and at 37 °C. 

#### 3.7.2. Cytotoxicity Assay

As directed by the manufacturer, the cytotoxic effect of EGA, blank EML-APA and the optimized EGA-EML-APA on MCF-7 cells was evaluated using the MTT viability test (ABCAM, Cambridge, UK). The cells were grown overnight at a density of 5 × 10^3^ cells per well in 96-well plates and further treated for 24 h with each treatment in a concentration range of 0.4 to 100 µg/mL. EGA was dissolved in DMSO (final concentration 0.1% *w*/*v*). The culture media were then aspirated and replaced with MTT solution at a final concentration of 2 mg/mL. The cells were subsequently incubated for 3 h at 37 °C. Thereafter, removal of the MTT solution was performed, then 100 μL of DMSO was added in order to dissolve the formazan crystals. Measurement of the absorbance was then carried out at a 570 nm wavelength using an Absorbance-based Spark^®^ multimode microplate reader (Tecan Group Ltd., Seestrasse, Maennedorf, Switzerland). The results were represented as a percentage of cell viability relative to the control. Fitting of the dose response curves followed by calculating IC50 values was undertaken using GraphPad prism software (GraphPad, Inc., La Jolla, CA, USA).

#### 3.7.3. Cell Cycle Analysis

Flow cytometry analysis was applied to analyze the effects of EGA, blank EML-APA, and the optimized EGA-EML-APA on MCF-7 cell cycle phases using propidium iodide (PI) Flow Cytometry Kit (ab139418, Abcam, Cambridge, UK). In addition, 3 × 10^5^ cells/well were seeded in a 6-well plate prior to their incubation for 48 h with each treatment. Subsequently, the cells were rinsed, trypsinized and then centrifuged for 15 min at 1000× *g*. The cells were then fixed using 70% ethanol and incubated at 4 °C for 2 h. The fixed cells were then centrifuged at 1000× *g* for 15 min prior to staining with PI (10 g/mL) and RNase treatment. A flow cytometer (FACScalibur, BD Bioscience, San Jose, CA, USA) was used to assess cell DNA content, with a minimum of 20,000 events obtained for each treatment. Analysis of the data was done using the CellQuest Software (Becton-Dickinson, BD, Erembodegem, Belgium).

#### 3.7.4. Apoptosis Assay

The Annexin V-FITC Apoptosis Detection Kit (BD Bioscience, San Jose, CA, USA) was used to investigate the effects of EGA, blank EML-APA and the optimized EGA-EML-APA on the apoptosis profile of MCF-7 cells. Briefly, in a 6-well plate, cells were plated at a density of 1 × 10^6^ cells per well and incubated for 48 h with each treatment. After that, the cells were trypsinized and subjected to centrifugation at 10,000× *g* for 5 min. The supernatant was then discarded; and the cells were rinsed in PBS and stained in dark conditions for 30 min using annexin V-FITC/PI dyes, as directed by the manufacturer. FACScalibur (BD Bioscience, USA) was used to evaluate stained cells, and a minimum of 20,000 events were obtained for each treatment.

#### 3.7.5. Mitochondrial Membrane Potential (MMP)

The determination of MMP was carried out as per the manufacturer’s directions using the MitoProbeTM TMRM Assay Kit for Flow Cytometry (Thermo Fisher Scientific, CA, USA). MCF-7 cells culture was performed at a density of 1 × 10^5^ cells per well in 96-well plates. Cells were stained using 20 M tetramethylrhodamine, methyl ester (TMRM) following 48 h of incubation with EGA, blank EML-APA, or optimized EGA-EML-APA and then incubated for a period of 30 min at 37 °C. TMRM is a fluorescent, cell-permeant dye that creates a strong bright signal upon its accumulation in active healthy mitochondria of cells. TMRM buildup ends and the signal disappearing indicates that the mitochondrial membrane potential is lost during apoptosis. The cells were subsequently washed in PBS, loaded with live-cell imaging buffer and then analysed using flow cytometry (FACSCalibur, BD Bioscience, USA).

#### 3.7.6. Real-Time Polymerase Chain Reaction (RT-qPCR)

##### RNA Extraction 

Qiagen’s RNeasy mini kit (Qiagen, UK) was used to extract RNA from MCF-7 cells as per the manufacturer’s instructions. A Nanodrop spectrophotometer (ND-2000C, Thermoscientific) was used to confirm the concentration and purity of RNA. A ratio of A260 nm/A230 nm, not smaller than 1.8, and a A260 nm/A280 nm ratio, not smaller than 1.9, were detected in all RNA samples.

##### cDNA Synthesis and PCR Amplification

RNA was normalized between samples and reverse transcribed to complementary DNA (cDNA) as per the manufacturer’s instructions using the iScriptTM One-Step RT-PCR Kit With SYBR^®^ Green kit (BioRad, Hercules, CA, USA). Relative expression patterns of *p53*, *bcl2*, *bax casp3*, *tnfα* and *nf-κb* were done utilizing 10 ng of RNA template in a 50-mL reaction mixture of iScript one-step RT-PCR kit with SYBR^®^ Green mix using a 7500 Fast real-time PCR system (Applied Biosystems, Waltham, MA, USA). 

## 4. Conclusions

Face-centred central composite design has been successfully employed for optimizing EGA-EML-APA with minimized vs. and maximized absolute ZP. The use of APA in formulating the emulsomes was proposed to provide additional advantage of enhancing uptake of EGA by cancerous cells, in addition to the cytotoxic activity of bee venom components including apamin. The measured responses of the optimized formulation were 267.6 nm for vs. and −32.1 mV for ZP. The measured responses coincide well with the predicted ones, confirming the validity of the numerical optimization adopted in this study. The present work confirmed the increased percentage of cytotoxic activity and apoptosis of EGA against the human breast cancer cell line (MCF-7) after the formulation EML-APA. This was confirmed by observations in cell viability (MTT assay) as well as the changes in the expressions of apoptotic markers and inhibiting NF-κB activity. Arresting G2/M and S cell cycle phases by EGA-EML-APA is a superior finding with respect to the mechanism by which the new formulation is inhibiting MCF-7 cell proliferation. These data demonstrate that the proposed formulation could be a successful delivery platform to enhance the cytotoxicity of EGA against MCF-7 cells. Thus, our study established the in vitro efficacy of optimized EGA-EML-APA as a potential anti-breast cancer formulation. Further in vivo investigation of such formulation following administration via non-invasive para-enteral routes compared to intravenous and oral ones will be considered in future work. 

## Figures and Tables

**Table 1:** Fit statistics of EGA-EML-APA responses according to the best fitting model.

Responses	Model	Sequential *p*-Value	Lack of Fit *p*-Value	R^2^	Adjusted R^2^	Predicted R^2^	Adequate Precision	PRESS
**Y_1_: vs. (nm)**	Quadratic	0.0001	0.1448	0.9972	0.9936	0.9806	53.49	3218.55
**Y_2_: Absolute ZP (mV)**	2FI	<0.0001	0.1997	0.9640	0.9425	0.8605	26.28	93.61

Abbreviations: EGA, ellagic acid; EML, emulsomes; APA, apamin; VS, particle size; ZP, zeta potential.

**Table 2:** Independent variables’ levels in EGA-EML-APA experimental runs and their measured responses.

RUN Number	Independent Variables			Dependent Variables	
	PC: CH Molar Ratio (X_1_)	PC: TS Weight Ratio (X_2_)	APA Molar Concentration (X_3_, mM)	VS * ± SD (Y_1_, nm)	ZP * ± SD (Y_2_, mV)
1	6:1	2:1	1.5	601.8 ± 18.9	−21.9 ± 0.7
2	4:1	1.5:1	0.5	364.9 ± 11.2	−35.5 ± 1.5
3	6:1	1:1	1.5	453.1 ± 13.7	−20.8 ± 1.2
4	4:1	1.5:1	1.0	382.1 ± 9.8	−28.5 ± 0.9
5	4:1	2:1	1.0	423.9 ± 12.6	−28.3 ± 1.1
6	4:1	1.5:1	1.0	389.3 ± 11.1	−27.9 ± 1.3
7	4:1	1.5:1	1.0	387.6 ± 10.6	−26.9 ± 1.1
8	4:1	1.5:1	1.5	392.5 ± 12.5	−23.5 ± 1.2
9	6:1	1:1	0.5	311.1 ± 10.8	−35.8 ± 1.8
10	2:1	1:1	0.5	263.7 ± 7.9	−28.1 ± 1.2
11	2:1	2:1	1.5	264.8 ± 8.8	−18.9 ± 0.6
12	6:1	2:1	0.5	598.1 ± 14.9	−46.1 ± 1.9
13	2:1	1:1	1.5	356.8 ± 13.1	−27.3 ± 0.9
14	2:1	1.5:1	1.0	356.5 ± 12.1	−30.3 ± 1.4
15	6:1	1.5:1	1.0	534.4 ± 16.1	−31.1 ± 1.3
16	2:1	2:1	0.5	321.7 ± 10.5	−33.9 ± 1.5
17	4:1	1:1	1.0	308.7 ± 8.9	−28.7 ± 1.2

Abbreviations: EGA, ellagic acid; EML, emulsomes; APA, apamin; PC: Phospholipon 90 G; Cholesterol: CH; TS; Tristearin; VS, particle size; ZP, zeta potential; SD, standard deviation. * Data are expressed as mean ± SD of five determinations.

**Table 3:** Face-centred central composite design (FCCCD) coded and actual variables’ levels along with the desirability constraints of the responses applied for optimization of EGA-EML-APA.

Factors	Levels		
	−1	0	+1
X_1_: PC: CH molar ratio	2:1	4:1	6:1
X_2_: PC: TS weight ratio	1:1	1.5:1	2:1
X_3_: APA molar concentration (mM)	0.5	1.0	1.5
**Responses**	**Desirability constraints**		
Y_1_: Vesicle size (nm)	Minimize		
Y_2_: Zeta potential absolute value (mV)	Maximize		

Abbreviations: EGA, ellagic acid; EML, emulsomes; APA, apamin; PC: Phospholipon 90 G; Cholesterol: CH; TS; Tristearin.

## Data Availability

Data are contained in the article.
